# Collaborative treatment of huge intrathoracic meningoceles associated with neurofibromatosis type 1: a case report

**DOI:** 10.1186/s13019-015-0374-y

**Published:** 2015-11-10

**Authors:** Deog Gon Cho, Yong Jin Chang, Kyu Do Cho, Jae Taek Hong

**Affiliations:** 1 Department of Thoracic and Cardiovascular Surgery, St. Vincent’s Hospital, College of Medicine, The Catholic University of Korea, 93 Jungbu-daero, Paldal-Gu, Suwon, Gyenggi-Do 442-723 Republic of Korea; 2 Department of Neurosurgery, St. Vincent’s Hospital, College of Medicine, The Catholic University of Korea, 93 Jungbu-daero, Paldal-Gu, Suwon, Gyenggi-Do 442-723 Republic of Korea

**Keywords:** Thoracic meningocele, Mediastinal disease

## Abstract

**Background:**

An intrathoracic meningocele is a relatively rare disease, and it commonly accompanies neurofibromatosis type 1. Patients tend to have no symptom but if its size is too large and compresses a lung and neighboring organs, it needs shunt drainage or surgical resection.

**Case Presentation:**

Herein, we present the case of a 52 year-old female patient with huge intrathoracic meningoceles associated with neurofibromatosis type 1, who has complained about chest discomfort and dyspnea at rest. As for a preliminary treatment, a neurosurgeon had performed a cystoperitoneal shunt, but the symptoms continued and the size of mass and the amount of pleural effusion did not change significantly. Therefore, the huge thoracic meningoceles were successfully treated through the thoracotomic approach in combination with lumbar puncture and cerebrospinal fluid drainage.

**Conclusions:**

It is reported that double huge intrathoracic meningoceles associated with neurofibromatosis type 1 was successfully treated by a shunting procedure followed by thoracotomic resection with collaboration of a neurosurgeon.

## Background

An intrathoracic meningocele is a relatively rare disease, 60 to 85 % of all thoracic meningoceles are associated with neurofibromatosis type I (NF-1) [1]. In the majority of cases, meningoceles are small or asymptomatic, and regular follow-up with periodic imaging are recommended without surgical treatment. Most patients become symptomatic between 30 and 50 years of age. Surgical treatment is indicated only when the size of the meningocele rapidly increases or when patients are symptomatic due to the compression of surrounding structures by the meningocele [2]. Various surgical techniques through a posterior laminectomy, a costotransversectomy with/without additional spinal arthrodesis, or a thoracotomy can be applied according to the size, location and combined anomaly [3]. And a shunting procedure of large meningoceles can be a valuable alternative treatment option in patients with otherwise high operative risks [4]. We describe a case with double huge intrathoracic meningoceles associated with NF-1 collaboratively treated with cystoperitoneal (CP) shunt followed by thoracotomic total excision.

## Case presentation

**Fig. 1:**
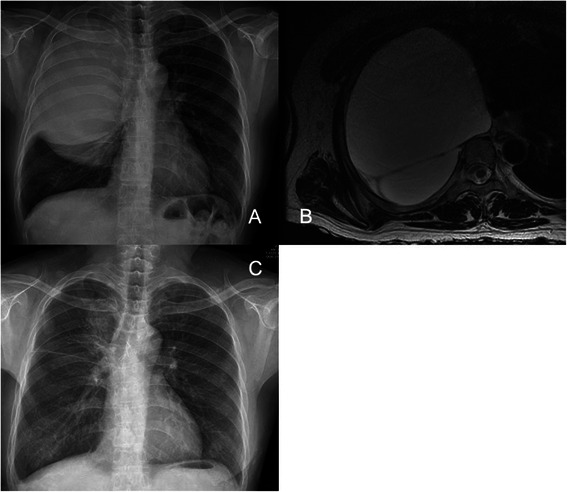
A plain chest X-ray (**a**) and magnetic resonance images show huge cystic masses on the right hemithorax continuing at the spinal canal (**b**). Post-operative plain chest X-ray (**c**) with a follow-up in 8 months after surgery: shows a markedly decreased meningocele of the posterior mediastinum and no sign of cerebrospinal fluid leakage

**Fig. 2:**
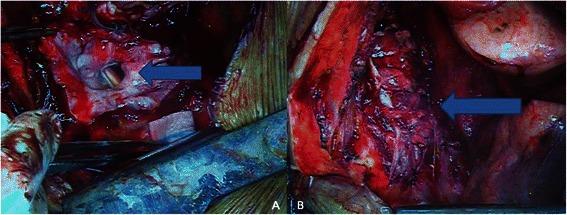
The root of meningocele, exposed spinal canal and the cord after excision of the meningocele (**a**, *arrow*). The remaing wall was carefully repaired and reinforced with the mediastinal pleura (**b**, *arrow*)

A 52 year-old female patient suffering from neurofibromatosis type 1 (NF1) complained about chest discomfort and dyspnea at rest. On past history, she underwent total thyroidectomy for nodular hyperplasia, and mass excision with skin flap operation for a huge sacral ulcerative neurofibroma. The plain chest X-ray showed a well-defined, huge cystic mass and compression of the right whole lung (Fig. 1a), and magnetic resonance imaging (MRI) revealed 14 x 11.5 x 11 cm, and 9.2 x 9.1 X 8.6 cm double cystic masses connected with T4-5 and T5-6 neural foramens in the right posterior mediastinum (Fig. 1b). We decided to do a surgical resection due to a symptomatic huge mediastinal mass. However, because this case had compression symptoms by a huge neurosurgical lesion, which could result in neurological problems or difficult manipulations during thoracic surgery, we performed a neurosurgical collaborative management. As for a preliminary treatment, the patient underwent the CP shunt procedure under general anesthesia by a neurosurgeon. A percutaneous catheter was inserted into the meningocele by transthoracic puncture with a needle at the T3-4 level under the fluoroscopy guidance, and a peritoneal catheter was placed through a limited abdominal incision. At postoperative 12 days, the CP shunt revision was performed due to accidental break of a cystic catheter. But the symptoms continued and the size of cystic mass and the amount of newly developed pleural effusion did not change significantly. We expected initial CP shunt would to perform functions to the patient, however, it was not sufficiently effective due to the thick wall of meningocele. Thus, surgical correction was decided and thoracotomy was the only choice since meningocele was extremely large to fill hemithorax. The CP shunt catheter was preoperatively removed at operative room, and then posterolateral thoracotomy was performed through the 5th intercostal space in the left down decubitus position. Two large meningoceles were compressing the whole lung out to collapse. After a dissection was conducted on the meningoceles including mediastinal pleura and a root of meningocele connected with neural foramen, and we suctioned spinal fluid (Fig. 2a). We checked the exposed spinal cord, and performed primly primary closure on the adjacent mediastinal pleura and some remaining meningocele walls without fluid leakage (Fig. 2b). The sealed area of the cystic wall was reinforced with an absorbable cellulose mesh and fibrin glue. CSF drainage with lumbar puncture was also performed to lower post-operative cerebrospinal pressure and to prevent spinal fluid leakage. The patient recovered immediately, and her symptoms of dyspnea improved after the surgery. Although CSF pressure was checked to be normal, there was CSF leakage in patient. Therefore we had kept lumbar drainage system for 5 to 7 days, 5 ~ 10 cc per hours. At postoperative 8 months, plain chest X-ray, CT and MRI revealed marked regression of the intrathoracic meningoceles and full expansion of the lung (Fig. 1c). There were no signs of spinal fluid leakage.

## Discussion

An intrathoracic meningocele is a cystic formation of the posterior mediastinum, originated by a saccular protrusion of the meninges in the thoracic cavity through the intervertebral foramen pathologically dilated, or by a bone defect in a thoracic vertebra [4]. The accepted etiopathogenesis is the dural dysplasia in patients with neurofibromatosis and enlargement of the intervertebral foramen [1]. In these patients the pleural traction through the negative intrathoracic force during inspiration and the pulsation of the CSF pressure would provoke evagination of the subarachnoid space through the intervertebral foramen [1]. Thoracic meningoceles are commonly detected in those aged in their thirties to fifties. They are also known to be slightly female predominant. However, there have been cases reported at ages ranging from two months to 73 years old [3]. Thoracic meningoceles should be differentiated from tumors, especially mediastinal tumors that commonly arise from the posterior mediastinum, such as neurofibroma, neuroblastoma, ganglioneuroma and posterior mediastinal cystic hygromas [2]. Multisequence and multiparameter magnetic resonance imaging plays an important role in the diagnosis, and defines the relationship between the cyst and the vertebral canal and nerve root or spinal cord [2]. If a patient has symptoms of a thoracic meningocele, surgical therapy can be considered [2]. Various surgical techniques can be applied according to the size of the cyst. In our patient with a hemithoracic meningocele and dyspnea at rest, we estimated the operative risk, and our collaborative team performed a shunting intervention between the meningocele and the peritoneum. However, the CP shunt was not sufficiently effective, therefore, we decided to perform secondary thoracotomic surgery. In case of the patient, there was no instability of spine, and since the meningocele was located at anterior portion of the spine, there was no related neurologic deficit. Also, we made a judgement that repairing with meningocele was enough, since it was thick. Recently, Chen et al. [5] reported the successful thoracoscopic plication for a huge intrathoracic meningocele associated with neurofibromatosis type I and kyphoscoliosis of the thoracic spine. In this case, primary CP shunt procedure performed, but shunt dysfunction was developed at postoperative 9 months later. Therefore, they performed thoracoscopic plication of cyst. We assume that this minimally invasive method could be an alternative valuable treatment of pulmonary intrathoracic meningoceles. In our case, we performed thoracotomic treatment of meningoceles due to double, wide and large defect of thoracic vertebrae. The approach of thoracotomy for intrathoracic meningocele removal or ligation is especially useful whenever a wider operative field is needed for complete resection of the cyst and anastomosis of the dura mater [3]. Although the shunt procedure can be a valuable alternative treatment option in patients with high operative risks [4], it can be ineffective or delayed control of patient’s symptoms, as in our case and other cases [5]. Therefore, we carefully recommend that a definitive adequate treatment should primarily be performed through thoracotomic or thoracoscopic approaches for large, symptomatic intrathoracic meningoceles in patients with acceptable surgical risks. Postoperatively, because CSF leakage is the most serious potential complication, we performed the reinforcement procedure of the suture site in the cystic wall, and CSF drainage with a lumbar puncture, which is helpful for maintaining constant intraspinal pressure.

## Conclusion

It is reported that double huge intrathoracic meningoceles associated with neurofibromatosis type 1 was successfully treated by a shunting procedure followed by thoracotomic resection with collaboration of a neurosurgeon.

## Informed consent

Written informed consent was obtained from the patient for publication of this Case report and any accompanying images. A copy of the written consent is available for review by the Editor-in-Chief of this journal. This case study was approved by Institutional Review Board for St. Vincent’s Hospital (VC14ZISE0245).
